# Multiparametric MRI for organ quality assessment in a porcine *Ex-Vivo* lung perfusion system

**DOI:** 10.1371/journal.pone.0209103

**Published:** 2018-12-27

**Authors:** Julius Renne, Marcel Gutberlet, Andreas Voskrebenzev, Agilo Kern, Till Kaireit, Jan Hinrichs, Patrick Zardo, Gregor Warnecke, Marcus Krüger, Peter Braubach, Danny Jonigk, Axel Haverich, Frank Wacker, Jens Vogel-Claussen, Norman Zinne

**Affiliations:** 1 Department of Interventional and Diagnostic Radiology, Hannover Medical School, Hannover, Germany; 2 Integrated Research and Treatment Center Transplantation (IfB-Tx), Hannover, Germany; 3 Clinic for Cardiothoracic and Transplantation Surgery, Hannover Medical School, Hannover, Germany; 4 Department of Pathology, Hannover Medical School, Hannover, Germany; 5 BREATH, German Center for Lung Research, Hannover, Germany; University of Michigan, UNITED STATES

## Abstract

**Introduction:**

Ex-vivo lung perfusion (EVLP) is an emerging technique promising an expansion of the donor pool and improvements in the outcome after lung transplantation. Reliable biomarkers for local assessment of organ function in the EVLP system are intensely sought after. This study aims to evaluate the feasibility of multiparametric functional magnetic resonance imaging (fMRI) in an EVLP system in a porcine aspiration model.

**Material and methods:**

Seven female pigs were anesthetized and gastric juice was instilled in the right lower lobe bronchus to simulate aspiration. Left lungs served as control. Lungs were removed and installed in a modified EVLP system. In the 12-hour EVLP run three sequential MRI scans were performed. Oxygen-washout time, Fourier Decomposition derived ventilation and perfusion, and dynamic contrast enhanced imaging derived perfusion were calculated. P_aO2_:F_iO2_ ratio was determined and correlated. End-point histology and computed tomography served as control.

**Results:**

All animals completed the protocol. MRI structural images showed infiltrates in lungs after aspiration comparable to CT scans. Ventilation was significantly (p = 0.016) reduced while perfusion was increased (p = 0.016) in lungs after aspiration. Non-contrast dependent Fourier decomposition perfusion showed good correlation (R^2^ = 0.67) to dynamic contrast enhanced derived perfusion. Oxygen washout time was significantly increased (p = 0.016) in lungs after aspiration and showed a correlation with the P_aO2_:F_iO2_ ratio (R^2^ = 0.54).

**Conclusion:**

Multiparametric fMRI for local assessment of organ function is feasible in EVLP and detects alterations in lung function following aspiration with correlation to clinical parameters. fMRI may improve organ assessment in ex-vivo perfusion systems, leading to a better selection of segments suitable for transplant.

## Introduction

Lung transplantation is an established treatment for end stage lung disease but is limited due to the shortage of suitable organs for transplantation [1]. Among others, impaired organ function due to aspiration is one of the main reasons for rejection of lung allografts [1]. To overcome this problem, ex vivo lung perfusion systems (EVLP) have been developed to enable a longer observation period of the explanted organ and to give room for treatment [2–4] and organ quality improvement [5,6].

Organ quality parameters measured in the EVLP should give the information needed to decide which part of the lungs can be safely used for transplantation. Currently, besides visual inspection and bronchoscopy the assessment of organ function within the ex-vivo lung perfusion system device is limited to the measurement of vascular resistance, pulmonary compliance and partial pressure of oxygen in the blood (P_aO2_) under ventilation with 100% oxygen. Clinically, P_aO2_ is the most frequently used parameter. However, P_aO2_ determined in the EVLP is under discussion because it is known that values are different for cellular vs. acellular preservation solutions -especially when shunts are present—and these data do not accurately predict the outcome after transplantation [7,8]. Histopathologic assessment of small samples only shows focal information that might not be relevant for the whole organ. In contrast, vascular resistance and pulmonary compliance can only serve as a parameter for the whole lung, without segmental information [8]. Therefore, new biomarkers for the evaluation of the lung allograft function on a segmental basis within the EVLP system are needed.

The use of imaging has been suggested early in the era of EVLP lung systems [9] and it has been shown that computed tomography is reliably able to detect injuries and pathologies causing inferior organ quality in organs rejected for transplantation [10]. Furthermore, in several animal studies the use of computed tomography, which is the clinical gold standard for detailed analysis of the lungs, was described [11,12]. However, only morphological but not functional evaluation using imaging were described so far.

In recent years multiparametric magnetic resonance imaging (MRI) of the lungs has matured. Different techniques deliver local quantitative parameters for ventilation (e.g. hyperpolarized gases, fluorinated gases and oxygen-enhanced imaging) and perfusion (dynamic gadolinium-enhanced imaging) besides high-resolution morphological information (ultra-short echo time imaging) [13–16]. In the EVLP system contrast agents cannot be cleared easily from the system, which deteriorates repeated measures. Therefore, any administration of contrast agent should be avoided which excludes the established dynamic gadolinium-enhanced imaging methods. Recently, a novel method for imaging of ventilation and perfusion without the need for intravenous or gaseous contrast agents: Fourier Decomposition has been introduced [17]. Multiple images with a very high temporal resolution are recorded and afterwards—using the mathematical technique of a Fourier transformation—different frequencies can be extracted and displayed separately—similar to dividing a musical chord into its separate notes. By choosing the appropriate frequencies images with ventilation and perfusion information can be derived. This technique might be beneficial for sequential imaging of the lungs in the EVLP system, since no additional gadolinium is needed and repeated measurements are possible.

Current EVLP systems are not compatible with MR scanners, therefore changes to the EVLP system are needed to assure safe operation within the scanner room.

This study aimed to explore the feasibility of functional pulmonary MRI using a standard proton-tuned scanner and a modified, clinically used EVLP system. Furthermore, non-contrast dependent Fourier Decomposition derived perfusion was compared to established gadolinium-based perfusion imaging. To compare the established parameter of organ quality monitoring (P_aO2_) within the EVLP system to the MRI derived parameters, a previously reported and clinically relevant porcine model of gastric juice aspiration [11] was chosen.

## Material and methods

### Animals and surgical protocol

This study was approved by the federal animal welfare committee of lower saxony, Germany (LAVES: Lower Saxony State Office for Consumer Protection and Food Safety; Appl. No. 33.9-42502-04-14/1604). All animals were treated according to the local animal welfare regulations and received humane care.

The protocol for aspiration injury induction followed a previous publication by Meers et. al. [11]. In brief, seven female pigs of German Landrace were anesthetized (weight median 56.2 kg, range 51.8 kg to 63.4 kg). Gastric tubing was inserted and gastric juice was collected. To simulate aspiration an angiographic balloon catheter was guided under bronchoscopic view to the lower lobe bronchus of the right lung. The balloon was inflated to block fluid transmission into other lung parts. 50 ml of filtered gastric juice was administered into the bronchus distally from the balloon. After 2 hours, the balloon was deflated and the catheter removed. Both lungs were cold flushed with Perfadex solution (XVIVO Perfusion AB, Gothenborg, Sweden) and removed from the animal following standard procedures for organ transplantation.

### Ex-vivo lung perfusion and imaging protocol

Both lungs were then wrapped in foil to simulate intrathoracic dimensions and installed in a modified ex-vivo lung perfusion system container (OCS lung system, TransMedics, Andover, MA, USA) using full blood from the same animal and Steen solution (XVIVO Perfusion AB, Gothenborg, Sweden) for a total of 12 hours [18].

In this first approach to a combination of MRI and the EVLP system safe operation was the main goal. Therefore, the container was completely detached from the EVLP system and all metallic parts were removed. In this approach all of the equipment not labelled as MR safe was required to be outside the scanner room. For ventilation an MRI compatible respirator placed in the scanner room was used (Aestiva/5 MRI, Datex Ohmeda, Madison, WI, USA), to limit the length of the gas circuit. Perfusate tubing was elongated to reach outside the room. A peristaltic pump was used to ensure pulsatile flow, which is needed for perfusion detection with non-contrast dependend MRI sequences (CAPS, Fa. Stöckert, Germany). For de-oxygenation of the blood an oxygenator (Affinity Fusion, Medtronic, Ireland) was used and operated with a gas mixture of 5.5% CO_2_, 12% O_2_ and 82.5% nitrogen (2 l/min). Perfusate flow was 1.5 l/min, slightly below previously published cardiac output of pigs with comparable size [12]. The respirator was set to a rate of 10/min and 500ml tidal volume, matching the in vivo parameters during the aspiration phase.

**Fig 1:**
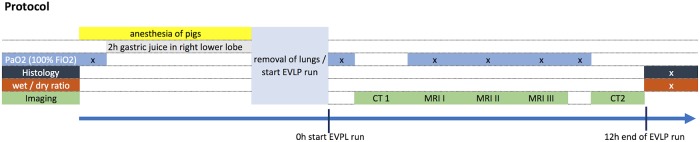
Experimental protocol.

All lungs were imaged using computed tomography and magnetic resonance imaging following the same protocol (Fig 1). In brief, after installation of the lungs in the EVLP system and a warm up period of 30 minutes with slow increase to the maintenance settings the first CT images followed by the first MR images were acquired. To get sequential data of the lungs within the EVLP System a second scan was administered at the middle of the EVLP run (i.e. after 6 hours). Last MRI scan was undertaken at the end of the EVLP run. The 2^nd^ CT scan followed the last MRI scan. Incidents in the scanner room were recorded, including mechanical damage to parts of the EVLP system or the MR scanner equipment and leakage of gas or fluids from the system.

### Imaging parameters

CT-scans of lungs as the clinical gold standard for structural lung assessment were acquired immediately before the first and after the last MRI using a 64-slice scanner (Lightspeed VCT, GE Healthcare, Milwaukee, WI, USA; slice thickness: 0.625 mm, tube current: 200mA, tube voltage: 120kVp).

Magnetic resonance imaging was performed on a 1.5 T scanner (Avanto, Siemens Healthineers, Erlangen, Germany).

The following sequences were used: ultra-short echo time (UTE)-sequence for structural assessment (TE = 0.07ms, TR = 6ms, FOV = 48 x 48 cm^2^, matrix size = 320 x 320, slice thickness = 1.5mm), an inversion-recovery snapshot fast low-angle shot sequence (IR Snapshot FLASH) [19] for oxygen dynamics using T1 mapping (TE = 0.8 ms; TR = 3.0 ms; acquisition matrix = 128x64; FOV = 50x50cm; slice thickness = 15 mm) under F_iO2_ = 0.21 and again, after 5 minutes of wash-in under F_iO2_ = 1.0. A half-fourier single shot turbo spin echo (IR-HASTE) sequence for oxygen washout calculation (TI = 6000ms; TE = 11 ms; TR = 661 ms; acquisition matrix 128x128; FOV = 45x45cm^2^; slice thickness 15 mm). For Fourier Decomposition analysis (i.e. ventilation and perfusion without the use of contrast agents), a half-fourier single shot turbo spin echo (HASTE) sequence was used (TE = 8.6 ms; TR = 661 ms; acquisition matrix 128x96; FOV = 50x50cm^2^; slice thickness 20 mm). Dynamic contrast enhanced imaging was performed using a clinically established time-resolved angiography with stochastic trajectories-volumetric interpolated breath-hold examination (TWIST-VIBE) sequence (TE = 0.74ms, TR = 2.55ms, FOV = 45x36cm^2^, acquisition matrix 224 x 127, slice thickness = 5mm). 1ml of 1:2 saline-diluted solution of Dotarem (Guerbet, Roissy, France) at 5cc/sec followed by 20ml of saline at 5cc/sec were injected into the arterial OCS connector. Total scan time for all sequences at each time point was about 60 minutes.

### Histopathological assessment

Samples of central and peripheral lung tissue were taken from the left and right lower lobes. Samples for histology were taken from identical localizations, fixed in buffered formalin 4% for 12-24h and embedded in paraffin according to standard histopathological protocols.

For histological evaluation of parenchymal damage and inflammation, 4 μm sections of formalin-fixed and paraffin embedded (FFPE) tissue samples stained with conventional hematoxylin-eosin (HE), periodic acid—Schiff (PAS) and Elastic Van Gieson (EvG) were used.

All samples were scored by a pathologist, experienced in pulmonary pathology using a four-point scale: 0 = none; 1 = mild; 2 = moderate; 3 = severe with regard to the following items: alveolar edema, hemorrhage, infiltration of neutrophils, pleuritis, atelectasis, mucus retention and epithelial disruption (adapted from [11]).

### Blood gas sampling

After 5 minutes of ventilation with an F_iO2_ = 1.0 blood samples were collected from the left and right lower lobe pulmonary vein at following time points (Fig 1): 1. in the animal before installation of gastric juice, 2. after 2 hours of gastric juice before and after removal of the lungs from the animal, 3. together with first, 4. second, 5. and third MRI and 6. at the end of the EVLP run. Blood gas samples were analyzed using a blood gas analyzer (Radiometer, Denmark).

### Cytokine / Chemokine analysis

Bronchoalveolar lavage fluid from right and left lower lobe were sampled at the end of the EVLP run. Blood samples (EDTA) were collected at the time of the first and second CT scan, i.e. at the beginning and the end of the EVLP run. Bronchoalveolar lavage fluid as well as blood samples were then analyzed with a Milliplex Porcine Cytokine Magnetic Bead Panel-Multiplex assay (Merck Chemicals GmbH, Darmstadt, Germany) according to the manufacturer´s instructions.

### Image processing and evaluation

All images were rated as sufficient or insufficient with regard to artifacts and signal to noise ratio by two radiologists (7 and 6 years of experience in cardiothoracic imaging). Both lower lobes were scored on CT and MRI UTE images by both radiologist, blinded to the study protocol, for areas with consolidation, following a previously reported scoring system [20]: 0 = no consolidation, 1 = small consolidation, 2 = consolidation less than 50% of lung lobe, 3 = consolidation 50–75% of lung lobe volume, and 4 = consolidation more than 75% of lung lobe volume.

T1 maps and Ventilation and Perfusion maps were calculated using self-developed MATLAB-scripts (Mathworks, Natick, MA, USA) as previously reported [21,22].

Washout times *w*_*out*_ were estimated voxelwise by mono-exponential fitting of the temporal signal.

Parenchymal blood flow maps were calculated using the Osirix-plugin UMM Perfusion with a fast deconvolution algorithm [23].

Image analysis was performed on a dedicated workstation using the open-source software Horos (Ver. 2.0.0 RC5). Both radiologists segmented the parenchyma of the right and left lower lobe in consensus, sparing the great vessels on the acquired morphological sequence. These ROIs were then transferred to the T1 maps, Fourier Decomposition ventilation and perfusion maps as well as the parenchymal perfusion maps.

One central coronal slice at corresponding slice location was used for analysis.

### Statistical analysis

Variables were analyzed for normal distribution using the D’Agostino-Pearson omnibus normality test. Since some variables did not show a normal distribution, the Wilcoxon matched-pairs test was used for comparison. For group comparison, the Friedman test was used and—where significant—followed by Wilcoxon matched pairs test. For interobserver agreement kappa was calculated. A significant difference was accepted for p≤ 0.05. Data were analyzed using Prism V 6.0h for Mac (Graphpad, La Jolla, CA, USA) and JMP Pro 13 (SAS Institute GmbH, Böblingen, Germany). Results are given as median (m) and interquartile range (iqr) in the following notation: m[iqr], and bars are representing the median with the interquartile range.

## Results

All seven pigs completed the protocol. There were no incidents regarding the MR scanner or the EVLP system and image quality was rated sufficient for analysis for all sequences acquired by both radiologists.

### Pathologic assessment

**Fig 2:**
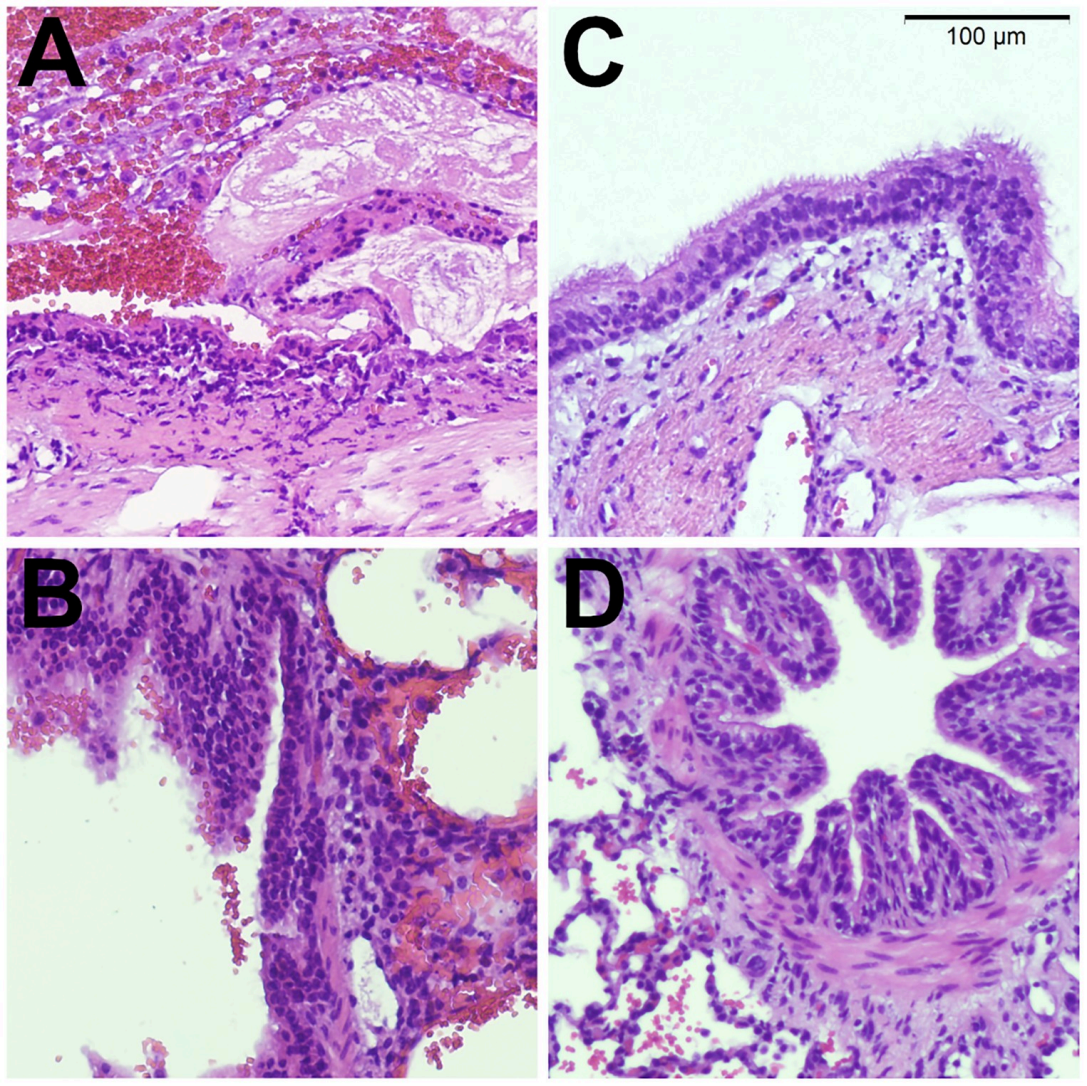
Histology. Representative histology images taken from a lung following experimental endobronchial aspiration (**A**,**B**). Large airways show extensive epithelial damage with necrosis and endobronchial hemorrhage (**A**). Distal airways have intact epithelium and show signs of hemorrhage, extending from more proximal sites into the alveolar space (**B**). Control lungs (**C**, **D**) show intact epithelium in large (**C**) and small (**D**) airways and focal intraparenchymal hemorrhage after ex-vivo perfusion (**D**).

**Fig 3:**
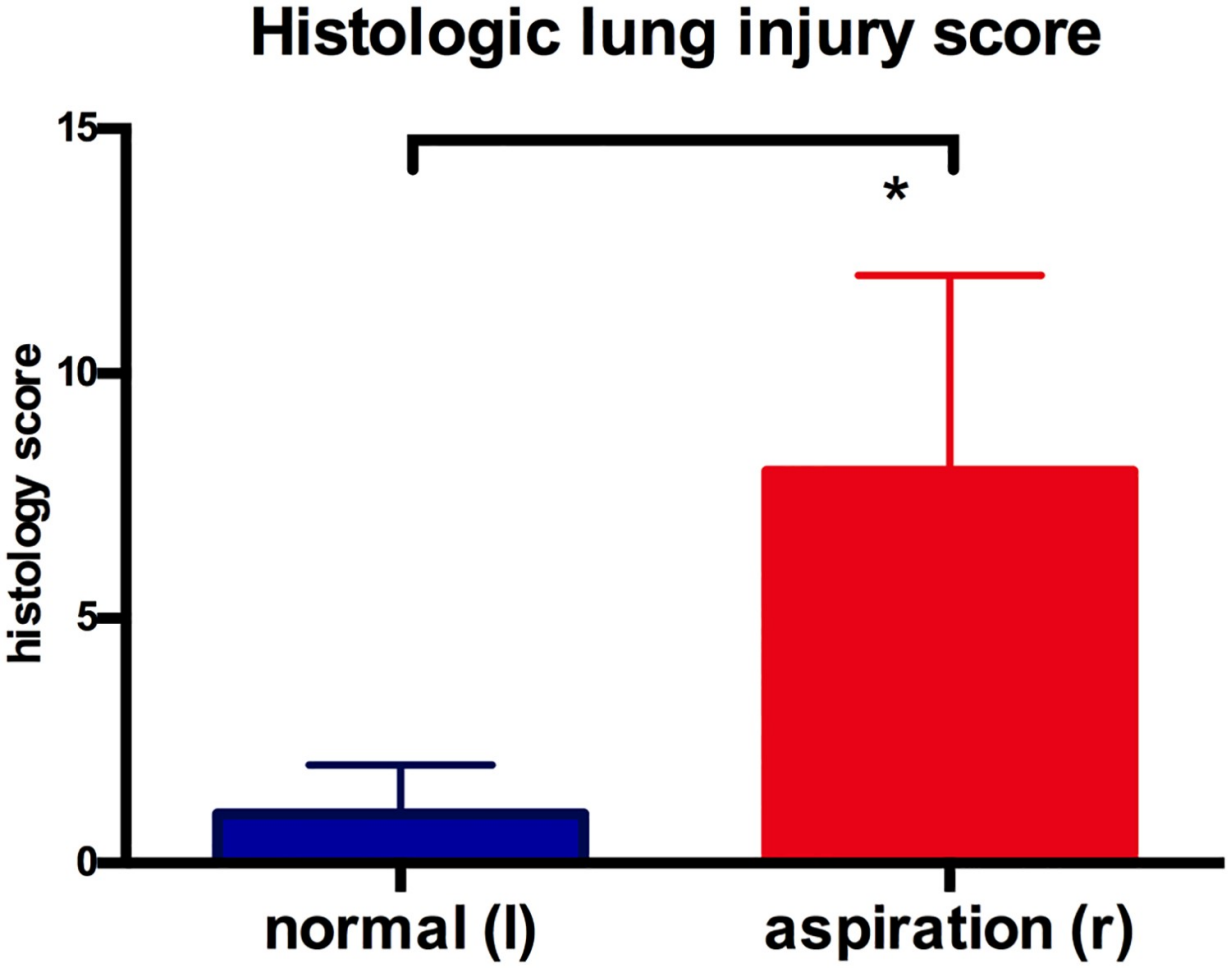
Histologic lung injury score. Histologic lung injury score was significantly higher (p = 0.016) for lungs following aspiration.

On gross evaluation, the right (aspiration) lung showed pronounced intraparenchymal hemorrhage. The left (control) lung showed minor focal parenchymal bleeding. Histology showed extensive epithelial necrosis, inflammation and hemorrhage in large airways of the right lung lower lobe (Fig 2). Small airways had predominantly intact epithelium, however hemorrhage retention of bronchial secretions could be observed. Histologic lung injury score was significantly higher in right lungs with aspiration, compared to normal left lungs (8 [4;12] vs 1 [0;5], p =.016; Fig 3).

### Blood gas analysis

**Fig 4:**
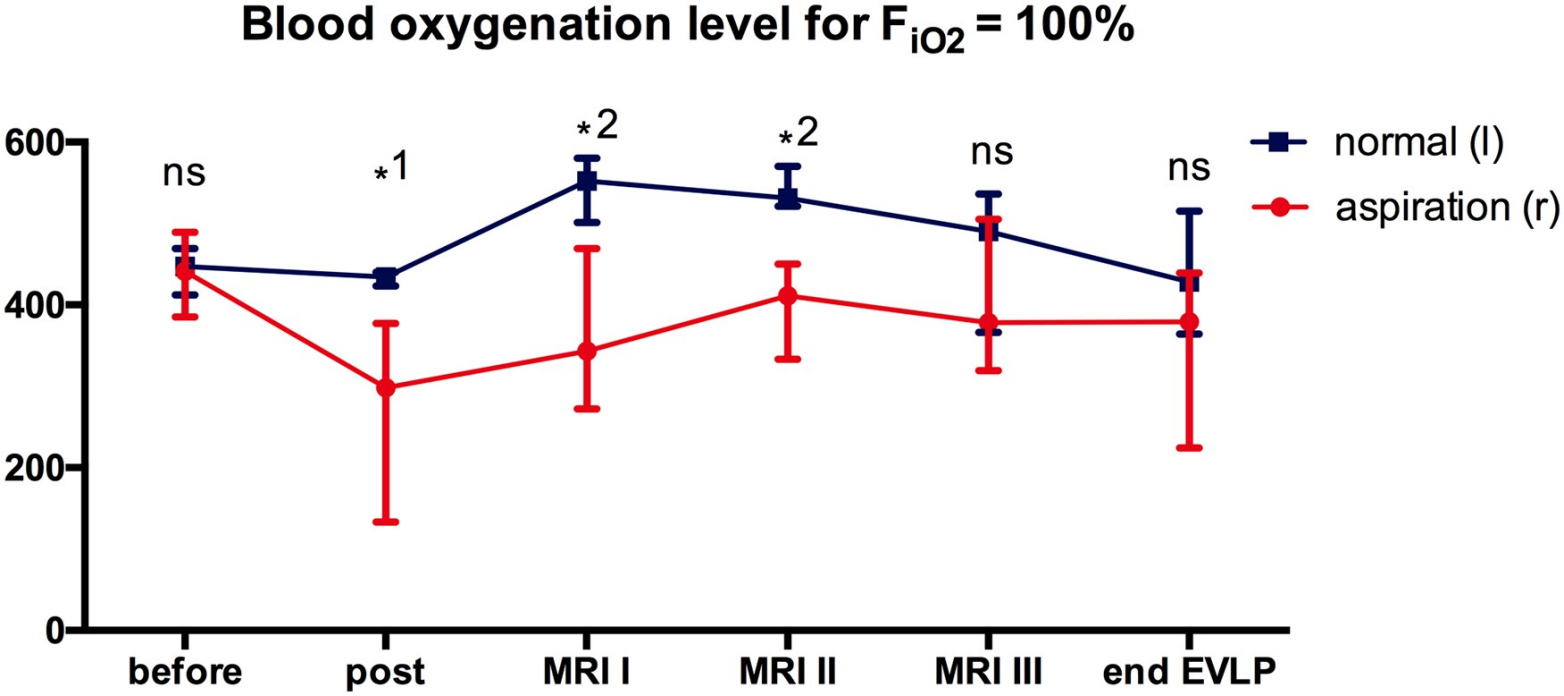
Blood oxygenation levels. P_aO2_:F_iO2_ ratio for FiO2 = 100% was determined in the right (aspiration) and left (normal) lower lobe pulmonary vein in the animal before and after 2 hours of gastric juice instillation as well as at MRI I, II, III, and at the end of the EVLP run. (ns not significant, *1 p = 0.031, *2 p = 0.016).

Blood oxygenation level (Fig 4) in the lower lobe pulmonary vein under ventilation with an F_iO2_ = 1.0 showed normal levels with no difference between right and left lungs before instillation of gastric juice (441[385;489] mmHg vs 447[412;469] mmHg). After 2 hours of gastric juice within the right lower lobe bronchus the oxygenation level decreased to 298[423;440] mmHg while the left lung remained stable (434[423;440] mmHg, difference between right and left lung p =.031). In the EVLP system there was a significant difference between left and right lungs at the time of the first and second MR (p =.016 and p =.016), with higher values for the left lung. There was an increase of blood oxygenation levels in the EVLP system, compared to the in-vivo levels after gastric juice installation (MRI I left lung: 552[501;580] mmHg, p =.031; MRI I right lung 343[273;469] mmHg, p =.156). Oxygenation level for the right lung remained stable until the end of the EVLP run (379[224;439] mmHg, compared to MRI I p =.688) while the left lung levels decreased (428[364;515] mmHg, compared to MRI I p =.047). At the time of MRI III and the end of the EVLP run no significant difference between right and left lungs could be observed (p =.109, p =.078).

### Chemokine / Cytokine analysis

There was a trend towards higher levels of proinflammatory cytokines including IL-1β (p =.031), Interleukin 6 (p =.063), Interleukin 8 (p =.031) and TNF-α (p =.063) in bronchoalveolar lavage fluid from lungs after aspiration, compared to normal control lungs. In contrast, levels were not significantly different between blood samples both at the beginning (IL-1β (p =.375), Interleukin 6 (p =.750), Interleukin 8 (p =.250) and TNF-α (p =.500)), as well as the end of the EVLP run (IL-1β (p =.469), Interleukin 6 (p =.813), Interleukin 8 (p =.438) and TNF-α (p =.156)). However, there was a significant increase for both the control lung as well as the lung with aspiration between the beginning and the end of the EVLP run (IL-1β (p =.016), Interleukin 6 (p =.016), Interleukin 8 (p =.031) and TNF-α (p =.016)).

### CT and MRI score

**Fig 5:**
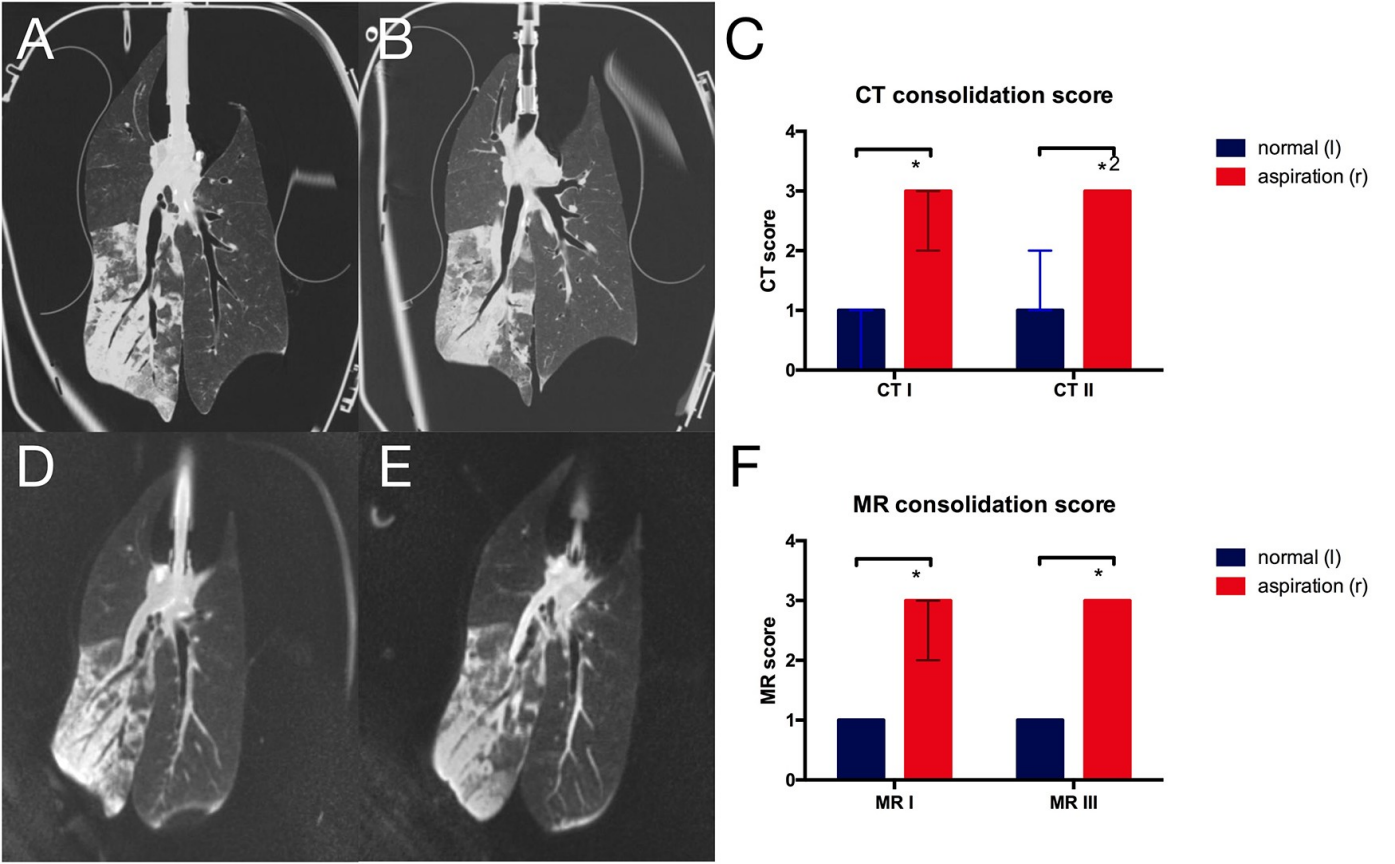
CT and MRI morphologic assessment of lung injury. Central coronal images of **A** first and **B** second CT scan as well as **D** first and **E** third MRI scan showing infiltrates after aspiration in the right lower lobe. Morphologic scoring of lung alterations based on **C** CT scans and **F** MRI scans. (*p = 0.016, *^2^p = 0.031; data from reader 1).

Both CT (Fig 5A and 5B) and MRI (Fig 5D and 5E) showed wide areas of consolidation in the right lower lobe, while in the left lung only small areas of consolidation or dystelectasis could be observed. The CT score for the extend of consolidation was therefore significantly higher for the right lower lobe on both scans (p = 0.016 and p = 0.031 for reader 1, p = 0.016 and p = 0.016 for reader 2, Fig 5C). Likewise, the MR-score was significantly higher in lungs after aspiration compared to normal lungs (p = 0.016, Fig 5F). There was no significant difference between the first and second scan for both MR and CT imaging. There was good interobserver agreement for the CT scoring (kappa = 0.73) as well as the MR scoring (kappa = 0.73).

### Oxygen-enhanced MRI

**Fig 6:**
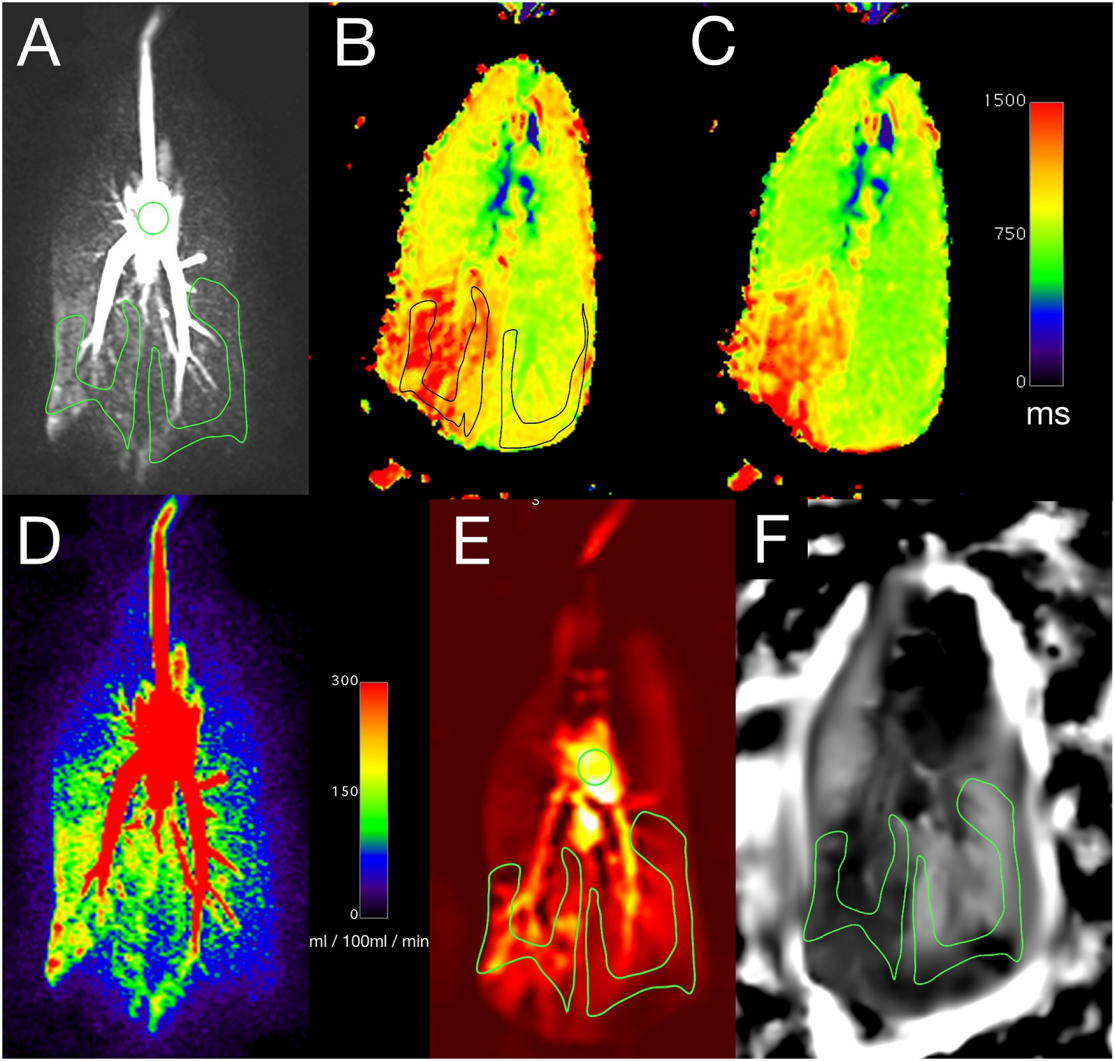
Functional MRI assessment. **A** Image of the dynamic contrast enhanced TWIST sequence with ROIs in the distal parenchyma of right and left lower lobe. The additional ROI in the central pulmonary artery was used for normalization of the perfusion weighted signal of Fourier Decomposition. Corresponding slices for oxygen enhanced T1 maps under **B** F_iO2_ = 0.21 and **C** F_iO2_ = 1.0. **D** Parenchymal perfusion map derived from DCE. **E** Perfusion weighted Fourier Decomposition with ROI placement and **F** Ventilation weighted Fourier Decomposition.

**Fig 7:**
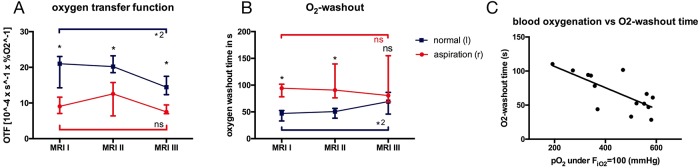
Oxygen-enhanced imaging quantification. **A** Oxygen transfer function (*p = 0.016, *2p = 0.031) and **B** oxygen washout time (*p = 0.016, *2p = 0.031) at all MRI examinations. **C** Correlation of P_O2_ with oxygen washout time at first MRI scan (R^2^ = 0.54, p = 0.003).

In the lungs with aspiration T1 relaxation times for both F_iO2_ = 0.21 as well as F_iO2_ = 1.0 were increased compared to normal control left lungs (Fig 6B and 6C). The oxygen transfer function (Fig 7A) was significantly reduced in lungs following aspiration compared to normal control lungs at first (9.06[7.07;11.63] 10^−4^/sec x P_O2_^-1^ vs 21.02[14.94;23.03] 10^−4^/sec x P_O2_^-1^, p = 0.016), second (12.57[6.37;15.74] 10^−4^/sec x P_O2_^-1^ vs 20.21[18.5;23.23] 10^−4^/sec x P_O2_^-1^, p = 0.016) and third MRI scan (7.53[7.01;9.45] 10^−4^/sec x P_O2_^-1^) vs 14.44[12.33;17.48] 10^−4^/sec x P_O2_^-1^, p = 0.016). While the OTF remained relatively constant on a low level for the lungs after aspiration, there was a decrease in the normal lungs (1^st^ vs 3^rd^ MRI p = 0.031).

In right lungs with aspiration, the oxygen washout time (Fig 7B) was significantly prolonged at first (94[78;102]s vs 47[33;53]s, p = 0.016) and second scan (91[76;140]s vs 51[38;57]s, p = 0.016). Towards the end of the EVLP run, the washout time for normal control lung was reduced compared to the first scan (p = 0.031), resulting in a not significantly different oxygen washout time at the last MR scan (81[69;155]s vs 69[46;86]s, p = 0.11).

There was a moderate correlation of the oxygen washout time with the blood oxygenation level under a F_iO2_ = 1.0 (1^st^ MRI: R^2^ = 0.54, p = 0.003, Fig 7C).

### Ventilation and perfusion

**Fig 8:**
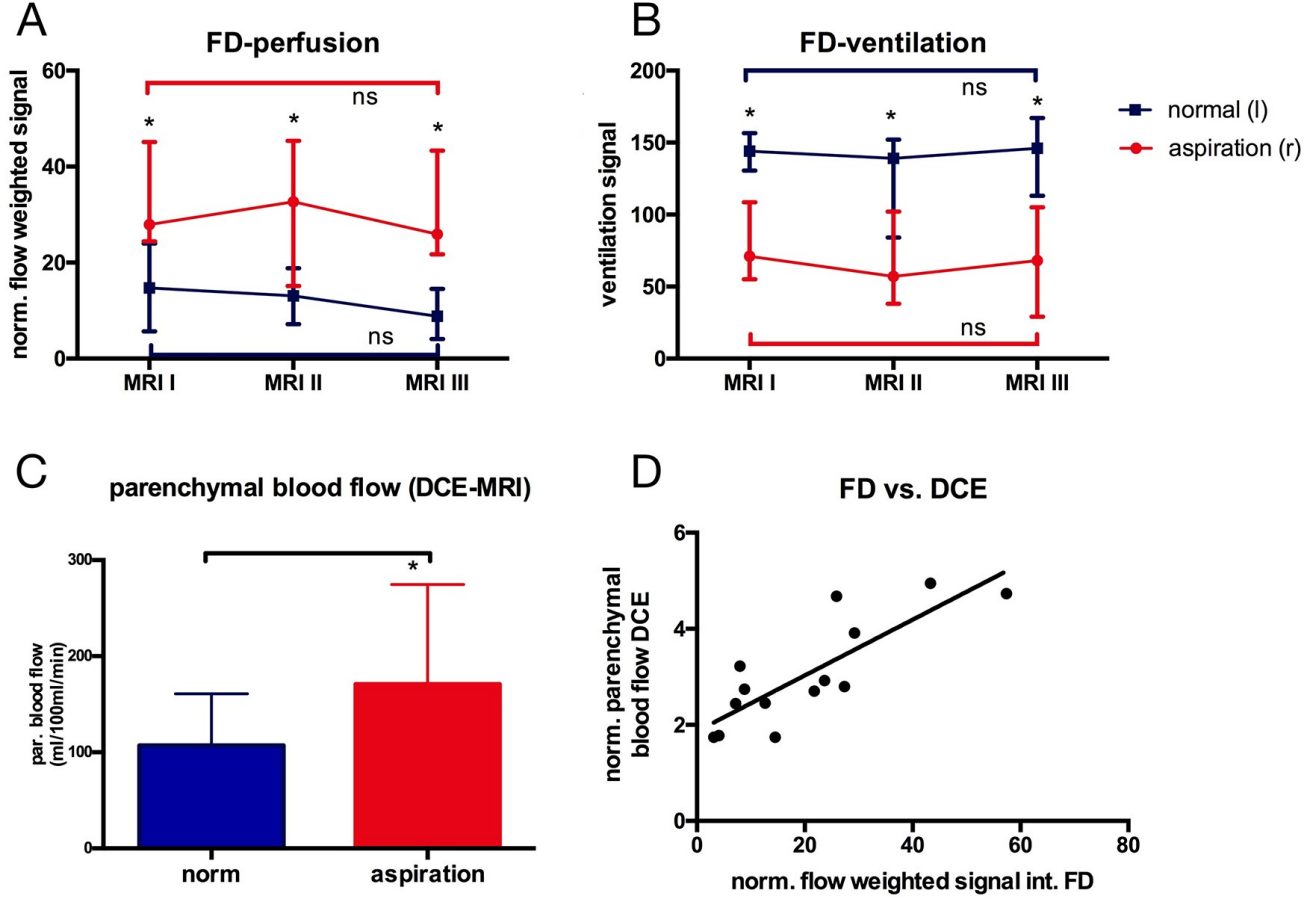
Ventilation and perfusion. Fourier Decomposition derived **A** normalized perfusion weighted (*p = 0.016) and **B** ventilation weighted signal (*p = 0.016). **C** Parenchymal blood flow derived from DCE-MRI (*p = 0.016). **D** Correlation of perfusion weighted Fourier Decomposition and parenchymal blood flow from DCE-MRI (R^2^ = 0.67; p = 0.001).

The normalized signal of the Fourier decomposition perfusion weighted images was significantly higher in lungs following aspiration compared to normal lungs (Figs 6E and 8A; MRI I: 27.9[24.5;45.1] vs 14.7[5.7;24.0] p = 0.016, MRI II: 32.7[15.1;45.4] vs 13.1[7.2;18.8] p = 0.016, MRI III: 25.9[21.7;43.3] vs 8.8[4.1;14.5] p = 0.016]. In contrast, the ventilation weighted signal was significantly lower in the lungs following aspiration (Figs 6D and 8B; MRI I: 57.0[54.0;105.0] vs 142.0[84.0;153.0] p = 0.016; MRI II: 57.0[38.0;102.0] vs 139.0[84.0;152.0] p = 0.016; MRI III: 68.0[29.0;105.0] vs 146.0[113.0;167.0] p = 0.016).

Dynamic contrast enhanced derived parenchymal perfusion at MRI III was significantly higher in lungs following aspiration (170.8[145.7;274.7]ml/100ml/min vs 107.2[89.6;160.8]ml/100ml/min; p = 0.016; Fig 8C). A good correlation between Fourier decomposition derived perfusion signal and dynamic contrast enhanced derived parenchymal perfusion was observed (Fig 8D; R^2^ = 0.67; p = 0.001).

## Discussion

This study shows the feasibility of quantitative functional MRI in an ex-vivo perfusion system. Non-contrast dependent Fourier Decomposition shows good correlation with established gadolinium-based perfusion imaging and therefore should be preferred in EVLP systems since no contrast administration is needed. High resolution MR images showed equal information to computed tomography scans. Reduced ventilation and increased perfusion as well as prolonged oxygen washout times confirmed the impaired lung function after aspiration and correlated with clinically established parameters.

The modifications of the EVLP system in this study were made with a maximum of protection against any damage to the MRI scanner as well as the EVLP system itself in mind. Therefore, the organ chamber was completely detached from the system and long tubing led to an MRI compatible respirator within the scanner room as well as to separate devices outside the scanner room, simulating the perfusion circuit of the EVLP system. However, only minor modifications to the EVLP systems enabling long tubings between the EVLP system and the container to enable a position of the system outside the 5 Gauss line within the scanner room should be sufficient for safe operation in future studies. For clinical studies the device needs to be declared as “MR safe” by the manufacturer.

Improvements to scanner hardware and post processing techniques lead to a steady increase in quality and resolution of magnetic resonance imaging [16]. In this study a sequence with ultrashort echo times was used and a resolution of 1mm^3^ was achieved. Since image quality was rated sufficient for diagnosis for all experiments and results for lung damage assessment were equal for both CT and MRI, previous data [24] on the power for detection of small pulmonary nodules may also be transferable to the assessment of lungs in the EVLP. As CT scans of donor lungs are not routinely available, this might add relevant additional information like tumor burden of the donor lungs when MRI is used in an EVLP organ assessment.

P_aO2_ under an oxygenation with an F_iO2_ = 1.0 is under debate for its accuracy as a parameter for lung function in the EVLP, as its values might not be comparable to in-vivo situations [8]. The levels observed in our study are comparable to previous publications using a porcine ex-vivo model [25], however in these studies only two hours of EVLP were used, reflecting the time of the first MRI in our setting where significant differences between injured and normal lungs were present. In other studies using different preservation solutions (but not an aspiration model) in a prolonged EVLP run over 24 hours a decrease in the P_aO2_:F_iO2_ ratio for certain preservation solutions over time in non-injured lungs was observed, comparable to the decrease observed in normal control lungs in this study [26]. Therefore, this decrease may be attributed to the EVLP function itself.

Elevated levels of proinflammatory cytokines in the BAL of lungs after aspiration and not in control lungs were observed. However, in blood samples taken from both lower lobes an increase of these cytokines was seen in both treated and control lungs. Therefore, the combined perfusion of injured and normal lungs may also have had an impact on the function of the normal control lungs. This is an important limitation of our study that should be addressed in future studies, preferably by treating lungs in one animal and using another animal without any treatment as control.

Although histology as well as computed tomography show the high extent of damage to the organ which is still present at the end of the experiments, there was no difference in P_aO2_:F_iO2_ at this time. Interestingly this is reflected in the oxygen washout time, resulting in a moderate correlation of these parameters. Given the stable impairment of both ventilation and perfusion which was observed in the Fourier-Decomposition analysis and the decrease in P_aO2_:F_iO2_ in normal lungs while constant on lower levels in the aspiration lungs this might reflect a slight decrease in the oxygen diffusion capacity in the whole lung due to slight edema formation in the EVLP system [27]. This might be attributed to the preservation solution used in this experiment, as previously reported [26], or again to the combined perfusion of normal lungs and lungs after aspiration.

As previously described, the oxygen transfer function is a marker dependent on ventilation and perfusion changes as well as on changes due to acute inflammation [28]. The constantly low level of the OTF in the aspiration lungs might therefore be attributed mainly to the inflammation present, while the decrease of the OTF in the normal control lung might again be attributed to the slight edema formation in the EVLP.

Fourier Decomposition is a new technique [17,29,30] delivering ventilation and perfusion weighted maps of the lung without the need of an inhaled or injected contrast agent. Since Fourier Decomposition is an analysis of time dependent signal changes [17], it is important to use a pump generating pulsatile and not laminar flow. The data of this study now confirm previous *in vivo* data [26] that Fourier Decomposition and DCE show a good correlation in the ex-vivo perfusion system. Therefore, Fourier Decomposition seems promising for analysis of perfusion in the EVLP system. Since Fourier Decomposition so far does not generate absolute values for parenchymal perfusion, the signal is weighted against the overall signal in the main pulmonary artery or inflow canula. This might explain the y-axis offset in the correlation with DCE (Fig 8D).

This study was intended as a pilot study to assess the feasibility of functional MRI in a small number of animals. Therefore, right and left lungs were used as injured and normal controls to minimize the number of animals needed. As a consequence, we were not able to measure vascular resistance and pulmonary compliance separately and therefore did not include these measurements in our study, which is a limitation. Furthermore, circulating cytokines from the injured lungs may also have had an impact on the function of the normal control lungs, since the perfusate flow was not separated in our setting as discussed above.

The extent of damage in the right lungs was relatively high, as reflected in the different injury scores on MRI, CT and histopathology. In future larger studies the use of different levels of lung damage is strongly needed to evaluate the sensitivity of the methods presented in this study. Because of the size of the study only global parameters were compared to MR imaging. The potential of local detection of lung injury needs to be further explored.

While MRI is a potentially cost intensive procedure in the already high cost surrounding of lung transplantation [31], the small numbers of lung transplantations performed annually overall and the ever-present donor organ shortage would easily justify the addition of a method—provided meaningful biomarkes can be derived. Local assessment of organ function within the MRI therefore might open the opportunity to expand the donor pool by targeted improvement of organs but also enables the assessment of lung segments separately, which might lead to an exclusion of just one lung segment from transplantation, which otherwise may have had an impact on organ function afterwards. This might increase the organ quality and long-term outcome of the recipient, granting the investment in the MRI. Furthermore, since all of the techniques applied in this work can be used on standard clinical MRI machines it should be readily available in all transplant centers worldwide.

Localized functional data from MRI measurements may also enable a more specific and sensitive treatment monitoring compared to morphologic assessment using computed tomography scans only. E.g. in pulmonary inflammation the amount of consolidation is not an early marker of treatment response, but may even increase in first place though the treatment is effective. Local information of oxygen transfer might be a better surrogate marker in this setting, but this has to be tested in future studies.

Beyond transplantation first efforts into new treatment strategies based on the use of EVLP systems for different end-stage lung diseases have been made. E.g. a recent study showed the feasibility of very high dose antibiotic treatment of lungs in an EVLP followed by autotransplantation [32]. Treatment for lung cancer in EVLP systems with high doses of cytostatic drugs or high radiation exposure are also thinkable. In all of these studies data of lung function with localized information would be beneficial and MRI may deliver these biomarkers.

In the setting of lung transplantation we suggest a comprehensive protocol using UTE for structural imaging, Fourier Decomposition for ventilation and perfusion analysis and oxygen-enhanced imaging for determination of oxygen-diffusion capacity impairments. This focused MRI examination with a total duration of about 20 minutes could be placed right after arrival of the donor lung within the EVLP system at the transplant center and might be repeated after interventions to evaluate the lung allograft prior to transplantation.

In conclusion, this study as a first step showed the feasibility of functional lung assessment using MRI in an EVLP system. Non-contrast dependent Fourier Decomposition for ventilation and perfusion analysis and oxygen-enhanced imaging with quantification of the oxygen washout time as a marker for alveolar function correlated with clinical parameters and should be combined with high resolution sequences for detection of lung nodules or infiltrates for a comprehensive protocol. This pilot study needs verification in larger trials and the next step should be a chronic model with transplantation in a recipient animal and correlation of the MRI derived biomarkers with the outcome after transplantation.

## Supporting information

S1 Data (XLSX)

## Abbreviations

DCE: dynamic contrast-enhanced imaging
EVLP: ex-vivo lung perfusion
FD: Fourier Decomposition
MRI: magnetic resonance imaging
OTF: oxygen transfer function
UTE: ultra-short echo time
